# Optimizing Clinical Trial Design to Maximize Evidence Generation in Pediatric HIV

**DOI:** 10.1097/QAI.0000000000001748

**Published:** 2018-07-11

**Authors:** Deborah Ford, Rebecca Turner, Anna Turkova, Martina Penazzato, Victor Musiime, Mutsa Bwakura-Dangarembizi, Avy Violari, Chishala Chabala, Thanyawee Puthanakit, Tavitiya Sudjaritruk, Tim R. Cressey, Marc Lallemant, Diana M. Gibb

**Affiliations:** * MRC Clinical Trials Unit at UCL, University College London, London, United Kingdom;; † HIV and Hepatitis Department, World Health Organization, Geneva, Switzerland;; ‡ Department of Paediatrics and Child Health, Makerere University, Kampla, Uganda;; § Research Department, Joint Clinical Research Centre, Kampala, Uganda;; ║ Department of Paediatrics and Child Health, University of Zimbabwe College of Health Sciences, Harare, Zimbabwe;; ¶ Perinatal HIV Research Unit (PHRU), University of the Witwatersrand, Johannesburg, South Africa;; # Department of Paediatrics, University Teaching Hospital, Lusaka, Zambia;; ** Department of Paediatrics and Child Health, University of Zambia, Lusaka, Zambia;; †† Division of Infectious Diseases, Department of Pediatrics, Faculty of Medicine, Chulalongkorn University, Bangkok, Thailand;; ‡‡ Division of Infectious Diseases, Department of Pediatrics, Faculty of Medicine, Chiang Mai University, Chiang Mai, Thailand;; §§ Research Institute for Health Sciences, Chiang Mai University, Chiang Mai, Thailand;; ║║ Department of Medical Technology, PHPT/IRD 174, Faculty of Associated Medical Sciences, Chiang Mai University, Chiang Mai, Thailand;; ¶¶ Department of Immunology & Infectious Diseases, Harvard T.H Chan School of Public Health, Boston, MA; and; ## Department of Molecular & Clinical Pharmacology, University of Liverpool, Liverpool, United Kingdom.

**Keywords:** clinical trial design, pediatric clinical trials, pediatric HIV

## Abstract

For HIV-infected children, formulation development, pharmacokinetic (PK) data, and evaluation of early toxicity are critical for licensing new antiretroviral drugs; direct evidence of efficacy in children may not be needed if acceptable safety and PK parameters are demonstrated in children. However, it is important to address questions where adult trial data cannot be extrapolated to children. In this fast-moving area, interventions need to be tailored to resource-limited settings where most HIV-infected children live and take account of decreasing numbers of younger HIV-infected children after successful prevention of mother-to-child HIV transmission. Innovative randomized controlled trial (RCT) designs enable several questions relevant to children's treatment and care to be answered within the same study. We reflect on key considerations, and, with examples, discuss the relative merits of different RCT designs for addressing multiple scientific questions including parallel multi-arm RCTs, factorial RCTs, and cross-over RCTs. We discuss inclusion of several populations (eg, untreated and pretreated children; children and adults) in “basket” trials; incorporation of secondary randomizations after enrollment and use of nested substudies (particularly PK and formulation acceptability) within large RCTs. We review the literature on trial designs across other disease areas in pediatrics and rare diseases and discuss their relevance for addressing questions relevant to HIV-infected children; we provide an example of a Bayesian trial design in prevention of mother-to-child HIV transmission and consider this approach for future pediatric trials. Finally, we discuss the relevance of these approaches to other areas, in particular, childhood tuberculosis and hepatitis.

## BACKGROUND

HIV-infected children differ from adults in that they acquire HIV around the time of birth when their immune system is still developing; if untreated, they experience more rapid disease progression and early mortality. Before ART became available, half of HIV-infected African children died before their second birthday.^1^ Conversely, children's immune systems have greater potential to recover than those of adults, particularly if ART is started in early childhood.^2^

As in other diseases in children, availability of drugs lags behind adults. It is now recognized by regulatory agencies that when disease progression, drug response, and exposure response in children are similar to adults, efficacy can be extrapolated from adult trials and only dose-finding and safety studies are required for pediatric regulatory approvals.^3–5^ For these, the drug in question (and age-appropriate formulations) should be investigated across the entire age range because drug disposition in children changes substantially with age.^6^ In contrast to regulatory trials, strategy trials are used to evaluate different treatment approaches (eg, sequence of regimens for first-, second-, third-line, treatment simplification, and use of more pragmatic dosing) and focus on effectiveness (ie, efficacy in the real world). Strategy trials often bridge the gap from the data required for regulatory approval to the data needed to inform clinical use and guideline development and are usually phase III–IV randomized controlled trials (RCTs).

In resource-limited settings, there are also programmatic issues and costs, eg, it is difficult for programs to procure and maintain reliable supplies of a large number of pediatric formulations, and liquid formulations are often impractical. Effective and safe fixed-dose combinations, ideally as dispersible scored formulations, dosed according to World Health Organization (WHO) weight-band tables, are now widely recognized ideal for HIV-infected children.^7^ As in adults, once-daily dosing is preferable for caregivers; however, higher drug clearance in younger children can make once-daily dosing a challenging pharmacokinetic (PK) goal.^8^ Limiting pill burden is also important, but it can be difficult to coformulate complex molecules in small-size scored pills that can also allow for dose adjustments as children grow.

A recent paper by Penazzato et al^9^ discussed the bottlenecks to rapid development of new antiretroviral drugs and formulations for children and outlined some possible solutions to streamline drug development studies seeking regulatory approvals, including: (1) enrollment of adolescents in adult trials; (2) simultaneous enrollment of children into different age/weight bands in early PK studies; and (3) optimized use of all available PK data and PK modeling. We will complement this paper by focusing on designs for large trials evaluating different treatment strategies.

Over the past 15 years, pediatric HIV RCTs have played a major role in changing treatment guidelines and leading to implementation of life-saving treatment and clinical management for children living with HIV. In this paper, we review the literature on types of trials that can improve efficiency, minimize time and costs and, in turn, save patient and health care resources.

## OPTIMIZING TREATMENT FOR CHILDREN

Scientific questions about optimizing treatment for children can take many forms, and will differ depending on whether they are viewed from the vantage points of policy maker, health care worker or family, and individual child or young person. WHO and the Collaborative Initiative for Paediatric HIV Education and Research (CIPHER) recently used Child Health and Nutrition Research Initiative (CHNRI) methodology for setting priorities in health research^10^ to identify research gaps in pediatric and adolescent HIV from a range of global stakeholders. The top priorities included determining “the safety, efficacy, acceptability, pharmacokinetics, and optimal dosing of existing and new antiretroviral drugs and formulations for children,” improving adherence in adolescents, and evaluating novel treatment delivery systems in children and adolescents.^11^ Management and prevention of coinfections, particularly tuberculosis (TB), viral hepatitis, and bacterial infections, is another area where data in children are largely limited to PK data on drug interactions.

All these questions would benefit from more efficient study designs that can address multiple questions while minimizing the number of patients enrolled.

## DESIGNING EFFICIENT TRIALS

Below, we describe trial designs for pediatric HIV that illustrate how several questions can be addressed within a single trial. Most are relatively long-term trials and include added-value PK, basic science, and social science substudies (Table 1).

**TABLE 1.**
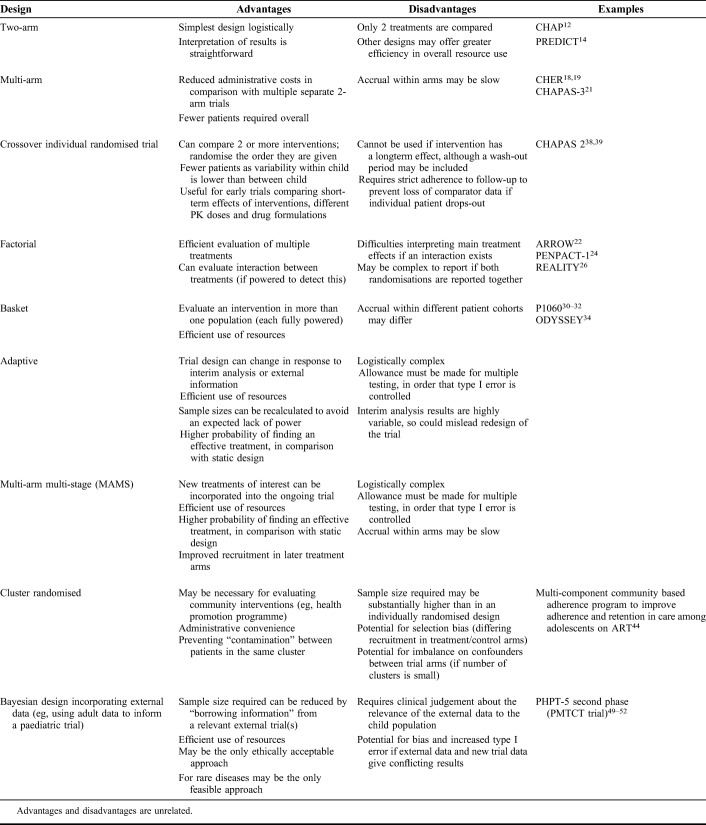
Advantages and Disadvantages of Different Trial Designs

### Parallel Two-Arm RCT

The Children with HIV Antibiotic Prophylaxis (CHAP) trial (2001–2003) was a classic double-blind randomized placebo-controlled trial in HIV-infected Zambian children.^12^ Five hundred forty-one children aged 1–14 years were randomized to cotrimoxazole prophylaxis or placebo (before available ART provision). The trial was stopped early because of a 43% reduction in mortality in the cotrimoxazole group and was followed by WHO guidelines recommending cotrimoxazole for all HIV-infected or exposed African children unless absence of HIV infection was demonstrated.^13^

The Thai PREDICT trial (2006–2011) also used a parallel randomized 2-arm design to compare early with deferred ART in children with CD4 percentage ≥15%.^14^ By necessity, PREDICT was “open-label,” with no placebo; although this is pragmatic and avoids unreal adherence, the issue of bias in such trials has to be considered when interpreting reporting of clinical endpoints.

### Parallel Multi-Arm RCT

Multi-arm trials offer advantages over 2-arm trials. They allow for more candidate treatments to be tested simultaneously and increase chances of finding an effective treatment in a shorter time frame.^15^ They are often more cost-effective than evaluating the same interventions in several 2-arm trials, by reducing the number of control patients needed and the administrative costs. Multiple hypotheses are tested within such trials; so, a well-defined analysis plan and appropriately estimated sample size are essential. A common approach is to power a multi-arm trial based on one global test across all treatment arms (usually of any difference between arms); only if this is significant, other comparisons are tested.^16,17^

**FIGURE 1.**
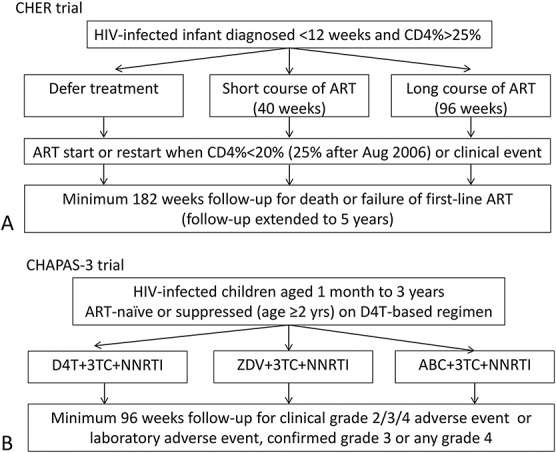
Parallel 3-arm RCTs: (A) CHER trial and (B) CHAPAS-3 trial.

The Children with HIV Early Antiretroviral Therapy (CHER) trial in South Africa was an open-label 3-arm RCT in 411 HIV-infected asymptomatic infants younger than 12 weeks recruited 2005–2007. Infants were randomized to deferred ART, immediate ART for 40 weeks (and then stop), or immediate ART for 96 weeks (and then stop) (Fig. 1A). ART was initiated in the deferred arm or restarted in the early treatment arms based on CD4 or clinical criteria.^18,19^ At the time of the trial, standard-of-care was deferred ART; the trial hypothesis was that early time-limited ART would be safe and provide long-term benefit by delaying immunological and clinical disease progression when compared with deferred ART. There was uncertainty around the optimal duration of early limited ART, with the possibility that 2 rather than 1 year of ART might allow for better development of the immune system, counterbalanced by increased risk of resistant virus with longer initial ART duration. A 3-arm trial allowed for both strategies to be evaluated together. The planned primary analysis was to test the null hypothesis of no difference among the 3 arms in time to death or treatment failure and only if the null hypothesis were rejected, to compare each of the early treatment arms to the deferred arm. Based on early CHER results,^18^ the data monitoring committee recommended that enrollment to the deferred arm be stopped, and children in that arm were evaluated for need to start ART; the other 2 arms continued enrollment. Publication of these early findings resulted in WHO recommending immediate treatment for all infants with confirmed HIV infection in 2008.^20^ The trial continued over 5 years, and demonstrated sustained benefit of early ART with both experimental arms performing better than deferred ART and a nonsignificant trend toward better outcomes with 96 weeks compared with 40 weeks of ART.^19^

The CHAPAS-3 trial in Zambia and Uganda (2010–2014) was another 3-arm trial, powered for a toxicity primary endpoint comparing 3 nucleoside backbones in both ART-naive and virologically suppressed children on stavudine-based first-line ART (Fig. 1B).^21^

### Factorial RCT

**FIGURE 2.**
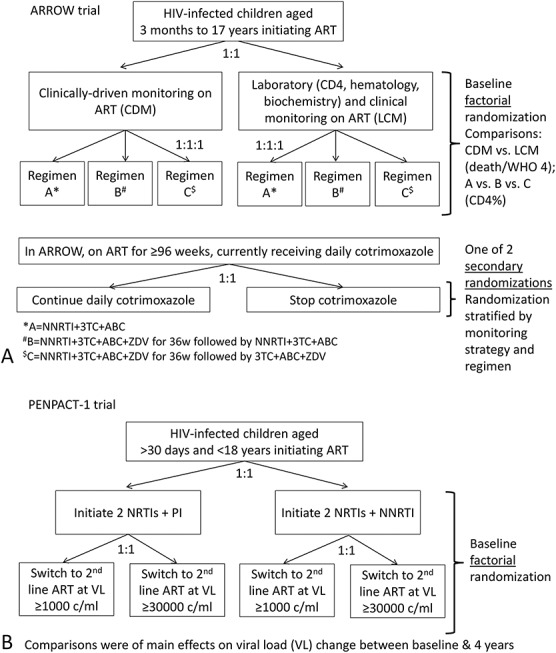
Factorial RCTs: (A) ARROW trial (B) PENPACT-1 trial.

A factorial trial simultaneously allows for 2 (or more) interventions, preferably targeting different aspects of a patient's disease, to be compared with standard treatment(s). ARROW (AntiRetroviral Research fOr Watoto) was a factorial trial evaluating 2 approaches for management of ART in 1200 symptomatic HIV-infected infants and children starting ART between 2007 and 2008 in Uganda and Zimbabwe. The first strategy compared clinically driven monitoring with routine laboratory monitoring plus clinical monitoring. The second strategy compared 3 different ART regimens (Fig. 2A).^22^ Children were randomized to both strategies at trial entry; thus, all 6 combinations (2 monitoring strategies × 3 regimens) were equally likely. As is common for factorial trials, at the design stage, separate sample size calculations were performed based on target effect sizes for the 2 strategies (using a noninferiority comparison for WHO 4 events/death for the monitoring arm comparisons and a global test of change in CD4% across the 3 ART regimens for the second strategy), with the larger of the 2 sample sizes taken forward.^22,23^ Critically, a factorial design usually assumes no interaction between interventions and, provided this is the case, significantly fewer patients are required than for a parallel multi-arm trial.^23^ In ARROW, although the predefined noninferiority criterion for clinically driven monitoring versus laboratory and clinical monitoring was met, CD4 monitoring provided a small clinical benefit after the first year on ART. Mean CD4 percentage did not differ between ART groups at weeks 72 or 144, although at week 36, participants on 4-drug regimens did better on average.^22^

PENPACT-1 was a collaborative 2 × 2 factorial trial sponsored jointly by the Paediatric European Network for Treatment of AIDS (PENTA) Foundation, Agènce Nationale de Recherche sur le Sida (ANRS), and the Pediatric AIDS Clinical Trials Group (PACTG), subsequently the International Maternal Pediatric Adolescent AIDS Clinical Trials (IMPAACT). Children initiating ART between 2002 and 2005 were simultaneously randomized to ART regimen (nonnucleoside reverse transcriptase inhibitor versus protease inhibitor + 2 nucleoside reverse transcriptase inhibitors) and to viral load threshold (1000 versus 30,000 copies/mL) for switch from first-line to second-line ART (Fig. 2B).^24^ Good long-term virological outcomes were achieved across the trial with no difference in mean viral load changes from baseline to 4 years by ART regimen or by switch threshold. The trial was not powered to detect interactions between regimen and switch threshold; however, for nucleoside reverse transcriptase inhibitor resistance, an interaction was observed with more major mutations in the group starting on a nonnucleoside reverse transcriptase inhibitor and switching to second-line at 30,000 copies per milliliter.

The recently completed REduction of Early mortaLITY (REALITY) trial included both HIV-infected adults and children aged 5 years and older (who have similar risks of disease progression by CD4 value after starting ART^25^) and was a 2 × 2 × 2 factorial trial (3 randomizations) evaluating 3 unrelated strategies to reduce mortality in patients starting ART with severe immunodeficiency in Africa.^26^ REALITY recruited only 72 children of 1805 patients with CD4 <100 cells/mm^3^, lower than expected because few children were found with CD4 <100 and adults recruited more quickly than anticipated.

### Secondary Randomizations After Enrollment

Another way to answer additional questions is to include secondary randomization(s) after enrollment, as in the ARROW trial. Children taking lamivudine and abacavir (3TC + ABC) containing first-line regimens were randomized after 36 weeks to continue twice-daily versus switching to once-daily 3TC+ABC^27^; in addition, children older than 3 years were randomized after 96 weeks to stop or to continue cotrimoxazole prophylaxis (Fig. 1A).^28^ Both randomizations were stratified by the 2 baseline factorial randomizations (described above). In this way, in addition to answering the original trial questions, ARROW also provided evidence for the first once-daily nucleoside backbone for children^27^ and for continued use of cotrimoxazole prophylaxis in children on ART.^28^

### Basket Trial

A basket trial includes 2 or more well-defined cohorts in which the same intervention is tested; the trial is powered to evaluate the intervention within each cohort.^29^ Efficiency is gained by simultaneous recruitment of the cohorts at the same clinical trial sites. The IMPAACT P1060 trial compared nevirapine with lopinavir/ritonavir (LPV/r) for first-line treatment in children younger than 3 years in Africa and India. Two cohorts were enrolled: cohort I included children with previous exposure to single-dose nevirapine for prophylaxis^30^; cohort II children had no previous nevirapine exposure.^31^ P1060 had a composite endpoint including both clinical progression and viral load response at 24 weeks. Recruitment to cohort I stopped early because results showed superiority of LPV/r^30^; cohort II completed planned enrollment and follow-up to 24 weeks and also demonstrated superiority of LPV/r^31^; follow-up continued for 5 years.^32^ WHO guidelines were changed in 2013.^33^

The ongoing ODYSSEY trial is also a basket trial including 2 individually powered, randomized phase III trials in ART-naive children and children failing first-line ART. Children are stratified based on ART history and then randomized to dolutegravir-based ART versus standard-of-care.^34^

### Adaptive RCT

The protocol for an adaptive trial prespecifies parameters (eg, dose, sample size, treatment, and patient selection criteria) that may be modified during the trial and the process for modification.^35^ Changes are planned and made “by design” preserving the statistical validity and integrity of the trial.^29,36^ Use of adaptive designs can lead to smaller sample sizes and increases chances of finding an effective treatment. The Multi-Arm Multi-Stage (MAMS) RCT is a particular type of adaptive trial in which the multi-arm element involves comparing several interventions simultaneously against a common control group; the multistage element requires patient recruitment to research arms that are not showing sufficient benefit to be discontinued, based on a series of preplanned, interim, futility analyses. It also allows for introduction of new research arms.^29,37^ MAMS trials may be appropriate where there are multiple drugs to be tested and where a surrogate short-term endpoint can be used to assess the likelihood of treatment success. MAMS trials have not, to the best of our knowledge, been used in pediatric populations, likely because most new drugs are initially evaluated in adults. They have also not been used in HIV, where long-term outcomes are usually required to assess efficacy.

### Individual Cross-Over Trial

**FIGURE 3.**
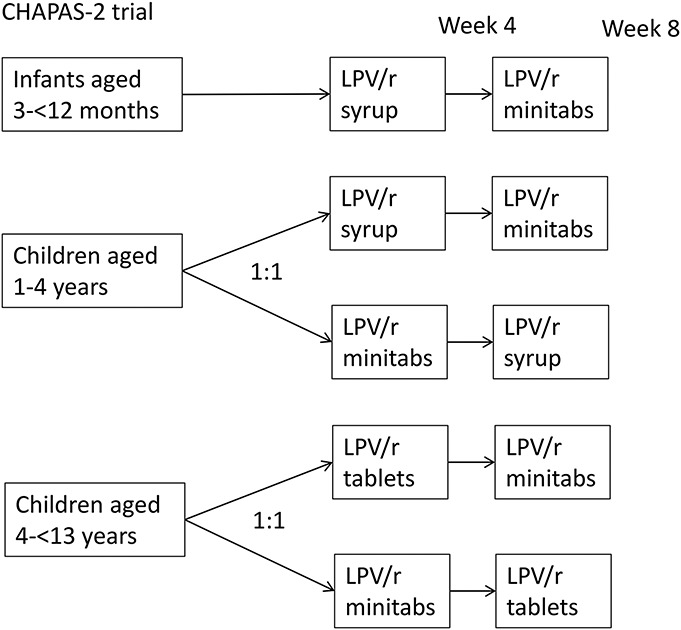
Individual cross-over trial: CHAPAS-2 trial.

A cross-over trial compares 2 interventions with each patient receiving both (the order of the interventions may be assigned at random) and is often more efficient than a parallel group trial because patient variation is removed from the treatment comparison. However, implementation is limited to considering short-term treatment effects on chronic conditions where it is appropriate for patients to receive both treatments. CHAPAS-2 compared PK and acceptability of different formulations of LPV/r in a cross-over trial of HIV-infected infants and children in Africa.^38,39^ In a 2-period cross-over design, children aged 3 months to 13 years had 4 weeks on one formulation of LPV/r and then switched to an alternative formulation. Order of formulation was randomized in the older age groups (Fig. 3). LPV/r exposure from minitabs was comparable with syrups but lower than from tablets.^38^ Follow-up to 12 months showed that preference for minitabs in younger children waned over time due to taste, although they were easier to store and transport than syrup.^39^

### Cluster RCTs

Cluster RCTs are used where the intervention is naturally applied to a group or cluster (eg, community, health centre, or school) and/or where individual randomization could result in contamination between the comparison groups (eg, sharing of medications in a household or learning about and implementing an experimental health prevention strategy in the control arm). Disadvantages are that a large sample size is usually required and there is a risk of selection bias if recruitment patterns differ systematically between treatment arms.^40,41^

Cluster RCTs are particularly helpful when investigating the impact of novel service delivery interventions, eg, to evaluate support strategies for women starting ART during pregnancy or breastfeeding (Option B+).^42,43^ An ongoing cluster RCT in Zimbabwe is evaluating a multicomponent community-based intervention to improve adherence to ART and retention in care of adolescents.^44^ A cluster RCT design is less likely to be appropriate for evaluating treatment for disease where the unit of intervention is the individual.

### Nested Substudies

Substudies can add significant value to a trial. The ongoing ODYSSEY trial^34^ includes 3 dolutegravir PK substudies: first, in children weighing less than 25 kg, which will complement the regulatory dose-finding study(IMPAACT P1093)^45^; second, in children weighing 25–40 kg, PK on both current recommended dolutegravir doses and after an increase to the adult dose of dolutegravir (also allowing for alignment with current ABC/3 TC fixed-dose combination); and third, dolutegravir and rifampicin PK in children with TB. There is also an immunology/virology substudy, a social science substudy, and a project to establish Youth Trial Boards to develop a model for adolescent patient representation in pediatric clinical trials.

## WHAT OTHER DESIGNS COULD BE CONSIDERED FOR HIV-INFECTED CHILDREN?

**FIGURE 4.**
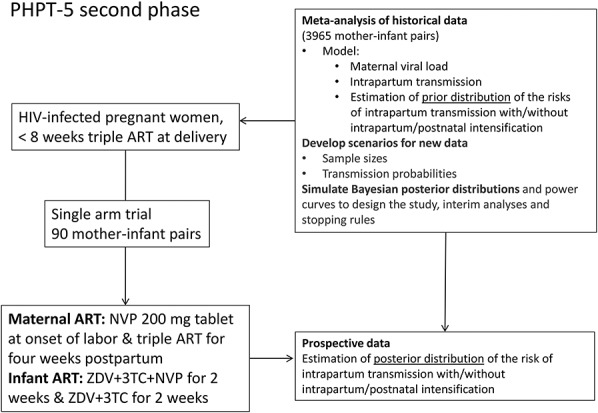
Bayesian trial design: PHPT-5 second phase.

Given decreasing numbers of vertically HIV-infected children globally, it is useful to consider trial designs used in rare and/or other pediatric diseases. Baiardi reviewed innovative designs for clinical trials in children, focusing on overcoming difficulties related to small sample sizes and ethical concerns^46^; other authors have proposed algorithms for choosing between different trial designs when researching rare diseases.^47,48^ An example is a recent trial of drugs for preventing intrapartum mother-to-child-transmission of HIV in Thailand that used a Bayesian approach to trial design.^49–52^ The trial's objective was to evaluate the efficacy of antiretroviral intensification, ie, adding maternal nevirapine during labor and infant triple ART prophylaxis in addition to standard-of-care in mothers presenting late in pregnancy. Design issues included ethical concerns about providing placebo where infants were at high risk and low numbers of mothers presenting late in the Thai context. Historical data from 3965 mother–infant pairs (enrolled in 3 previous randomized trials conducted in the same setting^49,53,54^) were used to build a model to generate prior distributions of intrapartum transmission probabilities with/without antiretroviral intensification. These probabilities were subsequently updated using results of a single-arm trial where 88 mothers and high-risk children received antiretroviral intensification; no transmission was observed (Fig. 4). The posterior probability of intrapartum transmission was 0.39% (95% CI 0.12–1.4) with intensification compared with 2.0% (0.3–5.2) without. The probability of superiority of intensification over standard-of-care (RR <1) was 0.94 and probability of at least 2-fold reduction of risk (RR <0.5) was 0.83. When solid historical data are available and equipoise cannot be assumed, a Bayesian design can provide confirmation where a standard design would not be appropriate.

In a similar way, a Bayesian design incorporating adult data could be useful for trials in HIV-infected children: by “borrowing information” from the evidence available in a completed adult trial and incorporating clinical judgment about the relevance of the adult data to the pediatric population; in this way, the sample size required in a pediatric trial could be reduced.^55–58^

## OTHER PEDIATRIC INFECTIONS

Coinfections with diseases such as TB, hepatitis, and bacterial infections occur in pediatric HIV and management of these children has generally been extrapolated from adults. Where logistically possible and scientifically appropriate, consideration should be given to including adults and older children and adolescents in trials together. Opportunities for PK substudies to evaluate drug–drug interactions in coinfected children within large pediatric trials are efficient and of low cost and should be encouraged.

As for HIV, scientific questions requiring larger trials in children with other diseases should be based on examining ways in which the disease differs in children compared with adults.

TB in children is paucibacillary in nature and unlike in adults, nonsevere forms predominate. Thus, although adult trials of treatment shortening have so far not been successful, this approach may be more likely to succeed in children with mild disease. The ongoing SHINE trial is a parallel 2-arm RCT comparing 4-month treatment with 6-month treatment in 1200 HIV-infected and HIV-uninfected children with nonsevere TB and will inform whether treatment shortening of drug-susceptible TB is efficacious and safe in this population^59^; it will also fill existing gaps in knowledge on dosing of new anti-TB formulations and commonly used HIV drugs.

Pediatric hepatitis C (HCV) has been neglected and there are no direct acting anti-HCV (DAA) drug regimens available for young HCV-infected children. The rapid development of multiple new drugs for adults from a number of innovator companies has meant that submitted pediatric investigation plans rapidly become out of date and very little has been realized in terms of evaluating age-appropriate formulations and dosing data of DAAs for children. To date, only 2 DAAs have been approved for adolescents aged 12 years and older.^60,61^ Now that several safe short regimens are available to cure the disease in adults, single-arm PK studies with safety data could be rapidly completed for the lead DAAs using a common protocol. However, an opportunity was lost to undertake an earlier adaptive trial comparing DAAs head-to-head in children, possibly including Bayesian approaches incorporating adult data as they became available.

Design of trials in TB, HCV, and hepatitis B has been complicated by difficulties with diagnosis: TB diagnosis is often presumptive and hepatitis C infection may clear in young children, making clinicians reluctant to treat before the third year of life. Hepatitis B, which is common (6%–10%) in Africa,^62^ may be more often acquired horizontally in childhood, and point-of-care E-antigen diagnostics, although under development, are not yet available. However, with the development of tenofovir alafenamide for HIV treatment, which is also an effective drug against hepatitis B, there may be opportunity to jointly evaluate tenofovir alafenamide for both diseases within the same trial in the future.

## CONCLUSIONS

Global research priorities for children and adolescents living with HIV highlight the remaining evidence gaps and the need to ensure that high-quality evidence is generated to inform clinical practice and global policies. This need, in the context of the changing epidemiology and decreasing resources, requires that innovative but rigorous trial designs are used when investigating key research questions. In this study, we have focused on describing the main types of trial designs for larger RCTs, with particular emphasis on increasing efficiency by addressing multiple scientific questions that cannot be extrapolated from adult trials. We have used examples of pediatric HIV trials to also demonstrate the value of embedded substudies and highlighted how similar principles should be used in designing trials in other infectious diseases in children and adolescents. Efforts should be made to ensure that innovative and efficient trial designs are funded. Combining innovation and capacity building will be the key to undertake high-impact research that will take us closer to reach treatment targets for children and adolescents living with HIV and other infections.
